# Survivorship care plan experiences among childhood acute lymphoblastic leukemia patients and their families

**DOI:** 10.1186/s12887-019-1464-0

**Published:** 2019-04-13

**Authors:** Samantha T. Pannier, Karely Mann, Echo L. Warner, Stephanie Rosen, Akanksha Acharya, Claire Hacking, Cheryl Gerdy, Jennifer Wright, Yelena P. Wu, Anne C. Kirchhoff

**Affiliations:** 1 Huntsman Cancer Institute, Cancer Control and Population Sciences, 2000 Circle of Hope, Salt Lake City, UT 84112 USA; 2 University of Utah, College of Nursing, 10 2000 E, Salt Lake City, 84112 USA; 3 Primary Children’s Hospital, Intermountain Healthcare, 100 N. Mario Capecchi Dr, Salt Lake City, UT 84113 USA; 4 Eli Lilly, 212 W 10th St # D180, Indianapolis, IN 46202 USA; 5 University of Utah Department of Dermatology, 30 North 1900 East, 4A330, Salt Lake City, 84132 USA; 6 Department of Pediatrics, University of Utah, P.O. Box 581289, Salt Lake City, 84158 USA

**Keywords:** Survivorship care plan, Treatment summary, Survivorship, Long-term follow up, Paediatric cancer

## Abstract

**Background:**

As survivorship care plan (SCP) use among childhood cancer survivors and their families has not been extensively researched, we report on their experiences with receiving an SCP after the completion of therapy.

**Methods:**

Eligible patients had acute lymphoblastic leukemia, completed therapy, and had no evidence of disease at enrollment. Patients aged 7 or older (*N* = 13) and at least one parent (*N* = 23 for 20 total patients) were surveyed and completed assessments at enrollment (Time 1, T1), SCP delivery (Time 2, T2), and follow-up (Time 3, T3) (retention 90.9%). Surveys assessed the delivery process and SCP format. McNemar tests were used to assess change from T2-T3.

**Results:**

Satisfaction with the SCP was generally high among parents. At T1 the majority of parents (69.6%) thought the SCP should be delivered after treatment but by T3 most preferred the plan to be delivered before the end of treatment (60.9%). While 95.7% of parents intended to share their child’s SCP with another provider, family, or school at T2, only 60.9% had done so by T3 (*P* < 0.01). At both T2 and T3, 100% of parents agreed that the SCP would help make decisions about their child’s future health care. Most patients at T3 (83.3%) felt they had learned something new from their SCP.

**Conclusions:**

Pediatric oncology patients and families feel SCPs are useful and will help them make decisions about health care in the future.

**Electronic supplementary material:**

The online version of this article (10.1186/s12887-019-1464-0) contains supplementary material, which is available to authorized users.

## Background

Survivorship care plans (SCPs) are an important tool for childhood cancer patients and their families as they transition into follow-up care after completing cancer treatment [1, 2], and are considered standard of care by leading cancer organizations [3]. However, delivery of SCPs is inconsistent. Multiple studies have found that few cancer patients receive an SCP, while at the same time there is limited evidence of SCP impact on health outcomes [1, 4]. Moreover, there are limited studies on whether childhood cancer patients and families find SCPs to be useful [5–7].

SCPs typically contain treatment summaries, information on diagnostic tests and results, tumor characteristics, supportive care (e.g. psychosocial and nutritional services) recommendations, information on follow-up care, and provider contact information [8]. Survivors of childhood cancer in particular could benefit from SCPs because they face transitions both from oncology to primary care and pediatric to adult cancer providers [9, 10]. Several studies have shown that childhood cancer survivors and their families have low levels of awareness and recall of their specific treatment and cancer experiences [11–13], as well as a limited understanding of cancer late effects [14, 15]. Comprehensive SCPs could provide key survivorship education and improve knowledge about treatment and health care after cancer.

Assessments of SCP delivery among adult cancer patients and adult survivors of childhood cancer have found that participants are receptive to SCPs, want detailed and individualized information, and use the SCP to communicate about their cancer care during other health care appointments [5, 16]. The transition out of oncology is a critical time for most patients and attrition in follow-up or confusion about the types of health care needed is not uncommon [17, 18]. SCPs may address some of problems encountered during the transition out of oncology. Due to the dearth of knowledge in this area for the childhood cancer population, studies of SCP delivery in pediatric oncology clinics are needed in order to develop best practices. Here we report on the experiences of patients and families receiving an SCP at a pediatric oncology clinic. Our goal was to determine parent and patient experiences with receiving an SCP, to understand whether they thought the SCP was useful and understandable, or whether it caused worry, and to identify their preferences for SCP delivery.

## Methods

The goal of this study was to implement an SCP program at Primary Children’s Hospital (PCH) in Salt Lake City, UT on a small scale, in order to obtain feedback prior to larger implementation of SCPs. We conducted this study in the outpatient hematology/oncology clinic at PCH, which serves as the main pediatric oncology clinic for the Mountain West. The University of Utah’s IRB approved this research. Written informed consent was obtained from all individual participants in the study including written assent from children ages 7 and older and parental permission for all patients under age 18.

We developed the SCP based on Passport for Care and American Society of Clinical Oncology’s SCPs [19, 20]. A patient and family advisory board, primary care providers [PCPs], and pediatric oncologists provided feedback on the plan’s layout and content. The SCP template draft was reviewed by our multidisciplinary research team [pediatric psychologist, health services researcher, pediatric oncologists, nursing director, oncology nurse coordinators, and health communications expert]. Prior to the launch of the SCP study, we conducted a focus group and surveys with oncologists and other providers to inform the SCP delivery process [21]. Feedback from these sources was incorporated into the design of the final SCP template and delivery process used in the present intervention. While our final SCP template generally contained similar content as Passport for Care’s SCP, we included additional information on vaccinations and health behaviors per our expert feedback. We also included QR codes that linked to Children's Oncology Group follow-up guidelines so that parents could easily access this material through their cell phones [22].

The final SCP template included: recommended medical and psychosocial follow-up, diagnosis details including cytogenetics, treatment information including protocol number, total doses of chemotherapy and radiation, vaccination history, oncology team contact information, preventive health behavior recommendations, educational web links on common survivorship issues, surgeries, allergies/adverse reactions, toxicities and complications, current or ongoing problems, and a section for additional physician’s notes. The SCP template is available as an online supplement (Additional file 1).

To develop the patient and parent surveys, we first reviewed and selected measures and domains from adult studies on survivorship care plans, as well as studies of pediatric survivorship care and survivor clinics [23–30]. We then employed the expertise of three pediatric oncologists, one oncology nurse, and a pediatric psychologist from PCH who reviewed the items and provided feedback. Following this step, a patient and family advisory board reviewed the outcome measure wording and provided feedback.

### Participant recruitment

Patients and parents for this study were recruited through chart review of post-therapy acute lymphoblastic leukemia (ALL) patients receiving care in the PCH outpatient pediatric hematology/oncology clinic from September 2015 to October 2016. As ALL is one of the most common cancer diagnoses in pediatric oncology, we focused on this disease as we anticipated being able to recruit adequate numbers that would help to inform SCP dissemination to other disease groups. Eligible families were English-speaking and had a child who completed therapy for ALL in the last 12 months. Eligible patients had to have no evidence of disease, be cognitively able to provide assent if age 7 and older, and be currently seen by one of eight pediatric oncologists participating in the SCP program. At PCH, off-therapy ALL patients are seen on average 5–6 times during a year over the first two years after the end of treatment. At least one parent, but sometimes both, participated in the study. Data collection was completed in May 2017.

### Procedures

Oncology nurse coordinators and pediatric oncologists shared responsibility for creating the SCPs. Prior to the start of the intervention, both groups were trained by the research team on preparation and delivery of the SCP. In the training, oncologists and nurses were provided with an electronic copy of the SCP template, copies of Children’s Oncology Group follow-up care guidelines, a protocol for the SCP delivery process, and instructions on how to fill out the SCP template including dose calculations for chemotherapy and radiation, and instructions on navigating the electronic medical record (EMR).

After a patient enrolled, a nurse coordinator completed 14 of the 17 sections in the SCP template including patient demographics, clinical factors, provider contact information, health education, and vaccine history. Once completed by nurse coordinators, the assigned pediatric oncologist reviewed the SCP and completed the final three sections (recommended follow-up care, preventive health behaviors, and case-specific comments).

Patients and families were surveyed at three separate time points: once at enrollment prior to the creation and delivery of the SCP (Time 1, T1), again immediately following delivery of the SCP approximately three months later (Time 2, T2), and a final time approximately three months after delivery of the SCP (Time 3, T3). To ensure that all patients and parents received a standard explanation of the plan, we created a standardized delivery script that was provided to the oncologists by the research team. The script directed the oncologist to review the concept of SCPs and to discuss key components of the patient’s SCP, and allowed the patient and family members to ask questions. Oncologists used these scripts when they delivered a completed SCP to patients and families at the T2 outpatient clinic visit approximately three months after enrollment in the study. The majority of parents and children completed their surveys at clinic visits, although a few T3 surveys were mailed if a clinic appointment was not scheduled within the follow-up period or the family moved.

### Data collection

Demographics were collected at enrollment from parents and included ZIP code of residence, allowing us to identify rural participants [31]. At each time point, we surveyed parents about their preferences about the SCP delivery process, and at T2 and T3 about their satisfaction and anticipated use of the SCP using 5-point Likert scales. At T2 and T3, we asked additional questions about their satisfaction with the SCP content.

Patients ages seven and older (*N* = 13) completed surveys that asked about comprehension of the SCP and the effects of the SCP on their level of worry. Patients under the age of seven were not asked to complete a survey. The survey data for parents and children included open and closed ended questions. All survey data were stored in REDCap.

### Analysis

We conducted data analyses using SAS v9.4 and Stata v14. Descriptive statistics were used to summarize demographics (e.g. insurance coverage, rural residence).

Five-point Likert scales for the parent survey responses were collapsed into binary categories (e.g., strongly agree and agree vs. neither agree nor disagree, disagree, strongly disagree). Child surveys were comprised of yes/no questions or write-ins. Write-in responses for both parent and child surveys were reviewed; due to the small number of responses, we did not conduct formal analyses but instead report write-in information as relevant. In families where both parents participated, parent surveys are reported independently of each other except in reporting child demographics where we report results from the parent who enrolled first in the study. The significance of the differences between individual responses to the parent survey at T2 and T3 were calculated using exact McNemar’s tests. All *p*-values are two sided and considered significant if *P* < 0.05

## Results

### Parent and patient characteristics

Of 35 families who met eligibility criteria during the study recruitment period, 23 were approached. A total of *N* = 22 families (N = 22 patients and *N* = 25 parents) were consented and enrolled (in 3 families, both parents requested to participate) for a 95.6% participation rate. One patient relapsed after enrollment and both the patient and parent were removed from the study sample. A total *N* = 21 patients and *N* = 24 parents completed T1. An additional patient and parent were lost to follow-up after T1. Overall, *N* = 20 families participated over the course of all three study time points for a retention rate of 90.9% (a total of 20 patients [13 of these who were ages 7 and older and surveyed] and 23 parents).

All parents were between 30 and 49 years old, 54.2% had a college degree, and most had an income of $60,000 or more (Table 1). Mothers comprised 75% of participating parents. Parents (91.3%) and patients (95.0%) were largely white and residents of urban areas (79.2%). All patients were insured (85.0% private, 15.0% public). Patients ranged from 3 to 18 years old and the largest group was between 7 and 12 years old at T1 (42.9%). Most were diagnosed with ALL ages 6 and under (76.2%).

**Table 1:** Parent and patient characteristics at T1

Parent (N = 24)^a^	N	%
Age at Enrollment, years		
30–39	19	79.2
40–49	5	20.8
Gender		
Male	6	25.0
Female	18	75.0
Race^b^		
White	21	91.3
Other	2	8.6
Ethnicity		
Non-Hispanic	21	87.5
Hispanic	3	12.5
Area of Residence		
Rural	5	20.8
Relationship to Patient		
Father	6	25.0
Mother	18	75.0
Level of Education		
High School Diploma/GED or less	4	16.7
Some College or Technical School	7	29.2
College Graduate or higher	13	54.2
Current Yearly Household Income		
<$59,999	9	37.5
$60,000 – 79,999	10	41.7
>$80,000	5	20.8
Marital Status		
Married or living as married	21	87.5
Divorced, separated, widowed, never married	3	12.5
Patient (N = 21)^a^	N	%
Age at Enrollment, years^c^		
3–6	8	38.1
7–12	9	42.9
13–18	4	19.0
Age at Cancer Diagnosis, years		
0–6	16	76.2
7–12	3	14.3
13–15	2	9.5
Gender		
Male	11	52.4
Female	10	47.6
Race^b^		
White	19	95.0
Other	1	5.0
Ethnicity		
Non-Hispanic	18	85.7
Hispanic	3	14.3
Insurance Type^d^		
Private	17	85.0
Public	3	15.0

^a^1 parent and 1 patient completed T1 surveys before being lost to follow-up

^b^Race missing *N* = 1 for both parent and patient

^c^Only patients 7 and older completed patient surveys

^d^Insurance type missing N = 1

### Parent preferences and experiences with receiving a survivorship care plan

Between T1 and T3, parents reported different preferences regarding when to receive an SCP, with 69.6% at T1 endorsing SCP delivery after treatment ended compared to 52.2% at T3 (Table 2). By the end of the study, many parents wanted their child’s SCP delivered before treatment ended (60.9%). Parents wanted an oncologist to deliver the SCP rather than a nurse at all three time points.

**Table 2:** Parent preferences and experiences with receiving SCP

*N* = 23	Time 1 (T1, enrollment) Pre-SCP		Time 2 (T2) SCP delivery		Time 3 (T3, follow-up) Post-SCP		T2 – T3 p-value
	N	%	N	%	N	%	
Preferences regarding							
When to receive SCP:^a^							
Before treatment ends	7	30.4	11	47.8	14	60.9	0.38
After treatment ends	16	69.6	13	56.5	12	52.2	>0.99
Provider to explain SCP to patient and families:							
Oncology Nurse	9	34.8	6	26.1	8	34.8	0.69
Oncology Doctor	18	78.3	18	78.3	19	82.6	>0.99
Experiences with SCP delivery and use							
SCP will have a very positive influence on communication with your child’s health care providers	b		16	69.6	12	52.2	0.13
Strongly agree to agree that:							
Provider used words that I could understand when explaining the SCP	b		23	100	22	95.7	>0.99
Provider made sure I understood everything on the SCP	b		23	100	22	95.6	>0.99
My provider answered all of my questions	b		23	100	21	91.3	0.50
Provider used medical terms without explaining what they mean	b		6	26.1	2	8.7	0.29
SCP contained all the information about survivorship I need	b		22	95.7	22	95.7	> 0.99
I feel concerned about the information I learned in the SCP	b		2	8.7	1	4.4	> 0.99
Very satisfied or satisfied with the:							
Process of receiving the SCP	b		22	95.7	22	95.7	>0.99
Layout of the SCP	b		22	95.7	22	95.7	>0.99
Page length of the SCP	b		21	91.3	22	95.7	>0.99
Content of the SCP	b		21	91.3	21	91.3	>0.99
Likelihood of using SCP over next:^c^							
6 months	b		19	82.6	13	56.5	0.07
1 year	b		20	87.0	15	65.2	0.13
2–4 years	b		21	91.3	17	73.9	0.22
≥ 5 years	b		20	86.9	19	82.6	>0.99
Recommendations regarding SCPs:							
Strongly recommend that SCPs should be used to other cancer survivors	15	65.2	20	87.0	19	82.6	>0.99
Strongly agree SCP will help me talk about health risks to my child in the future	14	60.9	13	65.5	14	60.9	>0.99
SCP will help make decisions about your child’s future healthcare	b		23	100	23	100	n/a

^a^Choices may add up to more than 100% participants could select multiple responses

^b^Question only asked at T2 and/or T3

^c^Likely/very likely vs. not/somewhat/moderately likely

When asked about their experiences with the SCP delivery and usefulness, at T2 69.2% and at T3 52.2% of parents reported that an SCP would very positively influence communication with health care providers. More than 90% of parents at both T2 and T3 agreed that the oncologist used appropriate wording when providing the SCP, made sure they understood the SCP, and answered all their questions. However, 26.1% of parents at T2 and 8.7% at T3 reported the provider used medical terms they did not understand (*p* = 0.29). By T3, most parents still felt the SCP contained all the information they needed (95.7%) and few were concerned about its content (4.4%). In the open-ended questions, one parent stated “*It helps me feel assured that my child will be monitored and cared for in the long term.*”

More than 90% of parents were highly satisfied with the process of receiving the SCP and its layout, length, and content at both time points (Table 2). At T2, approximately 80% to 90% of parents stated a strong likelihood of using the SCP over the next 6 months to 5 or more years. However, three months later (T3), they reported lower overall likelihood of future use at 6 months (T2 82.6% vs. T3 56.5%, *p* = 0.07) although over 80% still felt the SCP would be useful five or more years after receiving it. In addition, a few parents recommended in the write-in section of the survey that there was a need for additional details in the SCP. For example, one parent wrote that, “…*the drug and treatment (sections were) vague—want to know why drugs were given vs. other ones or side effects.*”

**Fig. 1:**
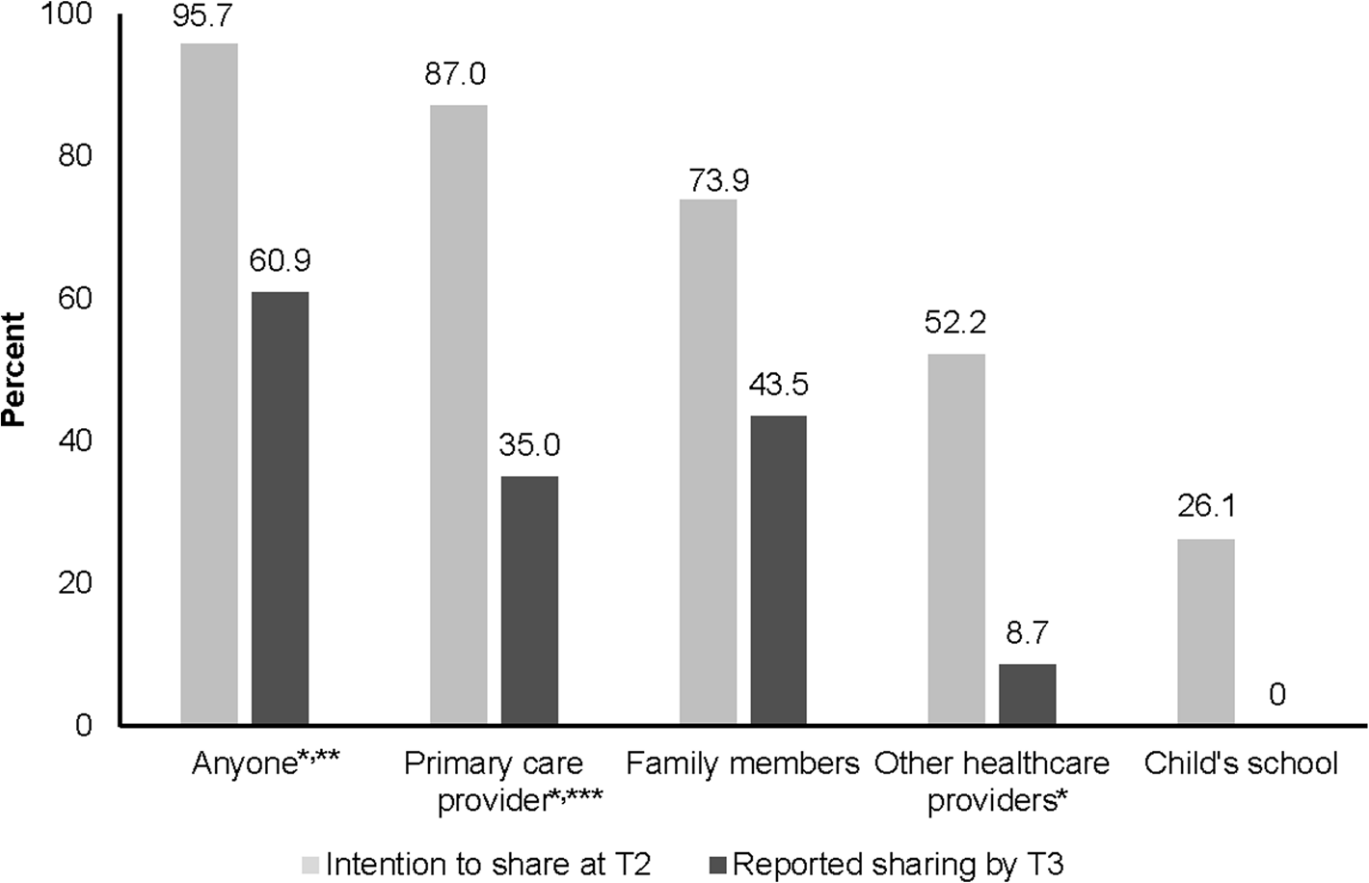
Parent intention to share SCP at delivery (Time 2) versus reported sharing at three months follow-up (Time 3). *P ≤ 0.01. **Any intention to share SCP (Time 2) or have shared SCP (Time 3). ***Time 3 limited to participants whose child had a PCP (*n* = 17)

At T2 delivery of the SCP (Fig. 1), 95.7% of parents intended to share their child’s SCP with someone (anyone included another provider, family, or school). However, only 60.9% had done so by T3 (*p* ≤ 0.01). More than half of the parents reported their intention to share the SCP with their child’s PCP (87.0%) or other family members (73.9%), but by T3 only a third had shared with a PCP (35.0%, *p* < 0.01, limited to the 17 patients who had a PCP) and only 43.5% with other family (*p* = 0.07). Approximately half of parents (52.2%) at T2 said they would share the plan with other health care providers, but at T3 only 8.7% had done so (*p* = 0.002). No parents had shared the SCP with their child's school at T3.

After receiving the SCP, parents were more likely to strongly recommend SCPs to other patients (65.2% at T1 compared to T2 87.0% and T3 82.5%, Table 2). Many parents strongly agreed the plan would help them communicate with their child about future cancer and treatment-related health risks (T1 60.9%, T2 65.5%, T3 60.9%). At both T2 and T3, 100% of parents agreed that the SCP would help make decisions about their child’s future health care. Some parents wrote in that the plan had a positive impact on their stress and hope for the future, as one parent stated “*It’s a comfort to have all that info in one place and easily accessible. Makes me feel more sure/positive/optimistic.*” While not directly asked, a few parents reported that they found a primary care provider for their child as a result of being in the study.

### Patient experiences with receiving a survivorship care plan

All surveyed patients (ages 7 and older) agreed it was important to keep seeing a doctor after finishing cancer treatment at all time points (Table 3). Few patients reported feeling worried about what they learned in the SCP at all time points. Two-third of patients (63.6%) at T2 reported that their physician used words they could understand when explaining the SCP while only 38.5% felt all their questions had been answered and 41.7% said the doctor used words without explaining what they meant. At T3, 83.3% of patients said they learned something new from receiving an SCP. When asked what they liked about the plan, one patient wrote that the SCP “*makes it easier for me to know what I need to get checked and easier for the doctor as well*.”

**Table 3:** Patient preferences and experiences with receiving SCP

N = 13	Time 1 (T1, enrollment) Pre-SCP		Time 2 (T2) SCP delivery		Time 3 (T3, follow-up) Post-SCP	
	N	%	N	%	N	%
Patient agreement with the following questions:						
It is important to keep seeing a doctor after I finish cancer treatment	13	100	13	100	13	100
I feel worried about what I learned about survivorship care plans	2	15.4	1	7.7	2^a^	16.7
An SCP will help me make decisions about my future health	b		13	100	12	92.3
My doctor used words that I could easily understand when explaining the SCP	b		7^a^	63.6	10^a^	83.3
During the visit my doctor made sure I understood everything on the SCP	b		12	92.3	11^a^	91.7
My doctor answered all my questions	b		5	38.5	2^a^	15.4
My doctor used words without explaining what they meant	b		5^a^	41.7	6	46.1
I learned something new from the SCP	b		b		10^a^	83.3

^a^One or more responses missing

^b^Question only asked at T2 and/or T3

## Discussion

While SCPs have been considered standard of care in cancer survivorship for over a decade, there have been few studies on the experiences of childhood cancer patients and families with SCPs. In this study, we found that most parents felt SCPs would be useful for managing their child’s health after treatment ended and liked the SCP content. Most parents planned on using the SCP both in the near-term and far into the future. However, they were less confident that the SCP would help them communicate with their child’s providers and their interest in using the SCP waned slightly over time.

Together, our findings demonstrate that at least in the initial months after delivery, families feel positive about SCPs and believe that they provide important information on health care after cancer, but that SCPs alone may not facilitate communication between families and providers. At T3, 52% of parents felt the SCP would have a positive influence on communication with their child’s providers, compared to 69% at T2 when the SCP was first delivered. Families may require coaching on when and how to use an SCP to communicate with other providers about their child’s cancer and to manage expectations at the end of treatment.

Furthermore, at SCP delivery, 87% of parents stated they would share the SCP with their child’s PCP, yet by T3 only 35% had done so. These findings suggest that cancer care teams creating SCPs should take the initiative to share SCPs with relevant providers as needed to ensure it is available as a patient transitions to primary care. Cancer care teams should be aware that part of this process may include helping families find a PCP to transition to if they do not already have one. Many families asked for digital copies of their SCP and integration with an online patient portal such as MyChart [6]. This may be an important avenue for improving SCP accessibility for patients, families and providers, although it was outside the scope of the original study.

Most parents preferred that the oncology doctor deliver the plan. For many families, oncologists are the most consistent provider seen during cancer treatment, which likely affects their levels of comfort and trust. However, in many institutions, nurses often coordinate late effects care, potentially suggesting that SCP delivery may need to be shared between both physician and nurse. In addition, approximately 60% of parents at follow-up in our study felt the SCP would have been useful to receive prior to the end of treatment. This could be a potential strategy to help patients and families begin the transition from treatment to survivorship, but would require additional resource support as the treatment summary sections of the SCPs would likely need updates once treatment ends.

Parents in general felt that the SCP was comprehensive, the delivery by the oncologist went well, their questions were answered, and they understood and felt positively about the SCP content. Yet 26.1% of parents at SCP delivery and 8.7% at follow-up reported that their child’s provider used medical terms they did not understand. While we created a standard script for oncologists to follow, our findings demonstrate that a small number of parents may need additional education on terminology and that oncologists should ensure that lay language is used to describe the SCP and to answer questions.

While parents remain primary decision-makers, ensuring that children with cancer understand their experience and know what care they need as they grow up is essential to managing their life-long health. Earlier studies have found that while adult survivors of childhood cancer can report their cancer diagnosis and generally know whether they received surgery, chemotherapy, or radiation, they are less likely to know the specific types of treatment received [11, 12]. While the children participating in this study spanned a wide developmental range (ages 7–18), over 40% at SCP delivery said their doctor used words without explaining what they meant, which is a communication gap that could be addressed through tailoring SCP discussions to the patient’s age.

Additionally, as children age, they should be provided updated information to ensure they transition into adult care informed of their follow-up care strategy. SCPs could be used to engage children in these discussions in a more complete and age-appropriate way. A few families reported to the research team that the SCP prompted them to find a PCP for their child. This suggests that the SCP could play an important role in assisting the transition from oncology care to primary care and follow-up care. During the study, parents occasionally requested new copies of the SCP as their children saw new primary care providers. Requests for corrections and additions (e.g., updated addresses, protocol number corrections) to SCPs were also not uncommon and came from parents and PCPs alike. Clinics implementing SCPs should be prepared for these future requests and consider developing appropriate policies on keeping patient records updated and adaptable. Clinics can also consider implementing tools such as Passport for Care available in the US and Survivorship Passport now available in Europe that allow for updating and accessing SCPs electronically [19, 32]. Survivorship care planning is an ongoing process and SCP templates should provide flexibility to adapt with pediatric patients as they mature.

## Conclusions

We had a limited sample size, which restricted our investigation of the full range of Likert responses and may have affected our ability to detect statistical significance across T2 and T3. Also, we restricted our study to ALL patients and so our findings may not be generalizable to other disease groups. Other cancer types may require additional detail or documentation in SCPs (e.g. cytogenetics of rare tumors, bone marrow transplant follow-up) and more in-depth discussions among providers, parents, and patients. We originally did not anticipate having more than one parent per child wanting to participate in the study. However, as childhood cancer affects the entire family, we opted to allow both parents to participate independently of each other if they both requested to participate. We suggest that future studies of SCPs in childhood cancer accommodate multiple participants within a family unit in data collection and analysis so as to inform survivorship support to the entire family [33].

While we tested the SCP delivery at a single site, we believe many of the experiences reported by parents and children are likely similar to other pediatric oncology clinics. In addition, long-term studies are needed to determine how SCPs affect survivors’ adherence to recommended follow-up care. A recent randomized trial that enrolled survivors of childhood cancer found that distribution of SCPs along with a directed PCP visit was not sufficient to improve adherence to guideline-recommended surveillance compared to survivors seen in a survivorship clinic [34], suggesting that more comprehensive efforts may be needed to get patients and providers to engage with SCPs. Finally, our sample was largely white, college educated, and English-speaking. Other institutions should take special care to design and deliver their SCP and delivery strategies in accordance with their population’s health literacy level as we found that a subset of parents did not understand some of the terminology.

In conclusion, we demonstrate that SCPs are an important tool for childhood cancer patients and their families at the end of cancer treatment. While only one piece of high-quality survivorship care, SCPs have the potential to serve as a fulcrum to transition patients from oncology care into survivorship care. At the same time, few parents in our study shared the SCP with other providers, demonstrating that oncologists may want to formalize delivery of SCPs to other providers. In future years, we hope to integrate SCPs into a new EMR system at PCH as informed by this study. Future studies should evaluate how parents and childhood cancer survivors use SCPs in the long-term after follow-up care ends and how SCPs influence access to appropriate risk-based survivorship care.

## Additional file

Additional file 1: “SCP Template no header.pdf” - Example Survivorship Care Plan template. (PDF 109 kb)

## Abbreviations

ALL: Acute lymphoblastic leukemia
PCH: Primary Children’s Hospital
PCP: Primary care provider (such as a GP, pediatrician, or family doctor/physician’s assistant/nurse practitioner)
SCP: Survivorship care plan
