# Supplementary material for: Survivorship care plan experiences among childhood acute lymphoblastic leukemia patients and their families
Source: BMC Pediatr. 2019 Apr 13;19:111. doi: 10.1186/s12887-019-1464-0 (PMC6461822; doi:10.1186/s12887-019-1464-0)
Supplement: Supplementary file 1 — “SCP Template no header.pdf” - Example Survivorship Care Plan template. (PDF 109 kb) [file 12887_2019_1464_MOESM1_ESM.pdf]

This is your Survivor Care Plan. It has information about your cancer and treatment. It also has recommendations for your follow-up care. This plan was made specifically for you based on your cancer treatment. Please share this summary with your doctors and also keep a copy for yourself.

**Name**:	**Sex**:	**DOB**:
**Date Created**:	**Cancer Diagnosis**:	
**Date Updated**:	**Completed by**:	

1. RECOMMENDED MEDICAL FOLLOW-UP

System	Recommended Tests/Screening	How Often Done	Date Last Done	Reason/Exposure
Heart				
Hormones				
Muscles/Bones				
Nerve/Brain Function				
Eye/Vision/Hearing				
Lung				
Fertility/Urination				
Second Cancer				
Other: __________				

2. RECOMMENDED PSYCHOSOCIAL FOLLOW-UP

If your child is having difficulty with any of the following:

- School or work performance
- Relationships or social interactions
- Emotions (such as worries, depressed or sad mood) or behavior changes

Please call Hematology/Oncology at [INSERT HOSPITAL/SOCIAL WORK PHONE LINE] and ask for a social worker, who can provide assistance or referrals to the appropriate professionals.

**Additional Resources:**	**QR Code**	**Guideline Applicable to Patient**
Education: http://www.survivorshipguidelines.org/pdf/healthlinks/English/educational_issues_Eng.pdf		☐
Emotional Needs: http://www.survivorshipguidelines.org/pdf/healthlinks/English/emotional_issues_Eng.pdf		☐

Recommendations are given by the Children’s Oncology Group and taken from their website http://www.survivorshipguidelines.org/

3. VACCINATIONS

Vaccination	Abbreviation or Common Name	Vaccination Completed	Date Vaccination Completed	Vaccination Follow-Up Dates if Needed
Tetanus, Diphtheria, Pertussis	DtaP	☐ Y ☐ N		
Haemophilus influenza type b	HiB	☐ Y ☐ N		
Polio	IPV	☐ Y ☐ N		
Hepatitis B	HepB	☐ Y ☐ N		
Hepatitis A	HepA	☐ Y ☐ N		
Pneumococcal Vaccine	PCV13, PPSV23	☐ Y ☐ N		
Measles, Mumps, Rubella	MMR	☐ Y ☐ N		
Chickenpox	Varicella	☐ Y ☐ N		
Influenza	Flu	☐ Y ☐ N		
Human Papillomavirus (HPV)	Gardasil	☐ Y ☐ N		
Meningitis	MCV	☐ Y ☐ N		

4. PROVIDER CONTACT INFORMATION (see other providers under section 8)

Provider Type	Name	Contact Information
Primary Care Provider		
Oncologist		

5. PROBLEMS OR CONCERNS

If your child develops a rash from low platelets or develops a prolonged fever (3-4 days) without any other signs of sickness you should contact your oncologist. For all other medical issues or questions, you should contact your pediatrician or primary care provider.

6. PREVENTIVE HEALTH BEHAVIORS

Type of Behavior	Recommendation	QR Code	Guideline Applicable to Patient
Diet and Physical Activity	http://www.survivorshipguidelines.org/pdf/healthlinks/English/diet_and_physical_activity_Eng.pdf		☐
Sun Protection	http://www.survivorshipguidelines.org/pdf/healthlinks/English/skin_health_Eng.pdf		☐

NOTE: These recommendations are based upon the current age of the patient. These recommendations will change over time. As the patient ages, they should refer to their primary care provider to receive instruction on more up to date recommendations.

Recommendations are given by the Children's Oncology Group and taken from their website http://www.survivorshipguidelines.org/

7. EDUCATION ON OTHER POTENTIAL HEALTH ISSUES

Potential Health Issues	Recommendation	QR Code	Guideline Applicable to Patient
Dental Issues	http://www.survivorshipguidelines.org/pdf/healthlinks/English/dental_health_Eng.pdf		☐ Y ☐ N/A
Fertility Issues	http://www.survivorshipguidelines.org/pdf/healthlinks/English/female_health_issues_Eng.pdf<br>http://www.survivorshipguidelines.org/pdf/healthlinks/English/male%20health_issues_Eng.pdf	(Female) (Male)	☐ Y ☐ N/A
Health Care	http://www.survivorshipguidelines.org/pdf/healthlinks/English/finding_and_paying_for_healthcare_Eng.pdf		☐ Y ☐ N/A
Heart Health	http://www.survivorshipguidelines.org/pdf/healthlinks/English/heart_health_Eng.pdf		☐ Y ☐ N/A
Secondary Cancer Risk	http://www.survivorshipguidelines.org/pdf/healthlinks/English/reducing_the_risk_of_second_cancers_Eng.pdf		☐ Y ☐ N/A
Other: ______________			☐ Y ☐ N/A
NOTE: If marked N/A recommendation not applicable.			
Recommendations are given by the Children's Oncology Group and taken from their website http://www.survivorshipguidelines.org/			

8. ONCOLOGY PROVIDER TEAM (see section 4 for oncology provider and primary care physician)

Provider Type	Provider Name	Contact Information
Nurse Coordinator		
Social Worker		
Radiation Oncologist		
Surgeon(s)		
Genetic Counselor		
Other: ___________		

9. CANCER DIAGNOSIS DETAILS

**EMPI #**:	**COG Reg #**:
**Date of Diagnosis**:	**Age at Diagnosis**:
**CNS Stage**:	**Date Therapy Completed**:
**Immunophenotype**:	**Other Sites Involved**:
**Diagnostic Details**:	
**Cytogenetics**:	**Last Evidence of Disease**:
**Medical History Prior to Diagnosis**:	
**Treatment Center**:	

RELAPSE(S)

**Date**:	**Site(s)**:	**Date Therapy Completed**:

SECOND CANCER(S)

**Date**:	**Type**:
**Stage/Site(s)**:	**Date Therapy Completed**:

10. CANCER TREATMENT PROTOCOL

Acronym/Number	Title/Description	Initiated	Completed	On Study
				☐ Y ☐ N
				☐ Y ☐ N

HEMATOPOIETIC STEM CELL TRANSPLANT

Infusion Date	Type	Source	Conditioning Regimen	Institution/MD

11. CHEMOTHERAPY/IMMUNOTHERAPY

Drug Name	Date of 1st Dose	Date of Last Dose	Cumulative Dose (mg/m^2)	Total Dose (mg)	Additional Information

12. SURGERY

Date	Procedure	Site (if applicable)	Side (right/left/both)	Surgeon/Institution

13. ALLERGIES / ADVERSE DRUG REACTIONS

Drug/Product	Reaction	Date

14. RADIATION

Site/Field	Laterality	Start Date	Stop Date	Fractions	Dose per Fraction (Gy)*	Initial Dose (Gy)*	Boost Site	Boost Dose (Gy)*	Total Dose (including boost) (Gy)*	Type

Radiation Treatment Center:

15. PAST COMPLICATIONS/TOXICITIES

Problem	Date Onset	Date Resolved

16. CURRENT OR ONGOING PROBLEMS

Problem	Date Onset	Date Resolved

NOTES
